# Comparison of safety and toxicity of liposomal doxorubicin vs. conventional anthracyclines: a meta-analysis

**DOI:** 10.1186/2162-3619-1-10

**Published:** 2012-04-23

**Authors:** Shamudheen M Rafiyath, Mohammad Rasul, Byung Lee, Guoqing Wei, Gurpreet Lamba, Delong Liu

**Affiliations:** 1 Division of Hematology and Oncology, New York Medical College and Westchester Medical Center, Valhalla, NY 10595, USA; 2 Bone Marrow Transplantation Center, the First Affiliated Hospital, Zhejiang University School of Medicine, Hangzhou, China

## Abstract

**Background:**

Liposomal formulations of anthracyclines appear to have favorable toxicity profile when compared with conventional anthracyclines in elderly, high risk cardiac patients and patients with prior use of anthracyclines. Randomized controlled trials have evaluated the efficacy and safety profile of liposomal formulations with conventional anthracyclines. Our aim is to evaluate the adverse effects and quantify the relative safety profile of the liposomal and conventional anthracyclines through meta-analysis of the published randomized trials.

**Methods:**

We conducted a broad search strategy of major electronic databases. We performed a meta- analysis of adverse effects on randomized controlled trials comparing liposomal formulation and conventional anthracyclines on different tumors. The primary outcome was the adverse effects including congestive heart failure (CHF), hematological toxicity, palmar-plantar erythrodysthesias (PPE), alopecia, nausea and vomiting. The odds ratios of the adverse effects were calculated separately and the overall odds ratio of the pooled data was calculated.

**Results:**

We identified nine randomized controlled trials comparing liposomal formulations and conventional anthracyclines. The study included 2220 patients, of which1112 patients were treated with liposomal formulations and 1108 were treated with conventional anthracyclines. We found that the liposomal formulations have low incidence of CHF(OR 0.34, 95% CI, 0.24–0.47), alopecia (OR 0.0.25, 95% CI, 0.0.10-0.62), neutropenia (OR 0.62, 95% CI, 0.45- 0.85),(OR 0.89, 95% CI, 0.71-1.125), and thrombocytopenia (OR 0.87, 95% CI, 0.61-1.25). The incidence of PPE was similar in both arms (OR 1.08, 95% CI, 0.11- 10.30).

**Conclusions:**

Liposomal doxorubicin and pegylated liposomal doxorubicin demonstrated favorable toxicity profiles with better cardiac safety and less myelosuppression, alopecia, nausea and vomiting compared with the conventional anthracyclines. The better therapeutic index of liposomal anthracyclines without compromising the efficacy makes it a favorable choice over conventional anthracyclines in elderly patients, patients with risk factors for cardiac disease and patients with prior use of anthracyclines.

## Introduction

Anthracyclines have become one of the most important drugs for the treatment of both hematological and solid tumors [1-3]. Conventional anthracyclines have a relatively low therapeutic index [4]. The risk of cardiotoxicity increases with higher cumulative doses of anthracyclines [4-7]. It is recommended that the cumulative life time dose of doxorubicin should not exceed 450–500 mg/m2. Anthracycline cardiotoxicity is an issue not only when it is administered as a single agent, but also when it is combined with other agents such as trastuzumab, which is a cardio toxic agent by itself [8]. The mechanisms for cardiotoxicity are mainly due to the development of cardiomyopathy as the result of free radical damage to the myocytes. The toxicity increases with high peak plasma anthracycline levels [9]. Repeated damage to the mitochondria of myocytes by the free radicals is believed to contribute to cumulative cardiomyopathy [10]. Several liposomal formulations of anthracyclines have been developed to increase the therapeutic index of anthracyclines.

The liposome-encapsulated anthracyclines was designed to reduce the toxicity of doxorubicin while preserving its antitumor efficacy by altering its tissue distribution and pharmacokinetics. Intravenously injected liposomes cannot escape the vascular space in sites that have tight capillary junctions, such as the heart muscle and gastrointestinal tract. The liposomes generally exit the circulation in tissues and organs lined with cells that are not tightly joined (fenestrated) or areas where capillaries are disrupted by inflammation or tumor growth. Thus, liposomes should preferentially direct doxorubicin away from sites of potential toxicity, but leave the tumor exposed [11]. Liposomal doxorubicin was associated with significantly less cardiac and gastrointestinal toxicity, while antitumor efficacy was at least comparable to that of the parent molecule [12,13].

Doxil/Caelyx is a Pegylated (polyethylene glycol coated) liposome-encapsulated (PLD) form of doxorubicin [4,7,14]. Doxil has preferential concentration in the skin because of the polyethylene glycol coating. The main dose limiting side effects associated with Doxil is the palmar plantar erythrodysesthesia (PPE), otherwise known as hand-foot syndrome. Following administration of Doxil, small amounts of the drug can leak from capillaries in the palms of the hands and soles of the feet. The result of this leakage is redness, tenderness, and peeling of the skin that can be uncomfortable and even painful. The prevalence of this side effect limits the Doxil dose that can be given as compared with doxorubicin in the same treatment regimen. Outside the United States, Doxil is known as Caelyx.

Myocet is a non-pegylated liposomal doxorubicin which is approved in Europe and Canada for treatment of metastatic breast cancer in combination with cyclophosphamide. The rationale behind its design is similar to Doxil [15-20]. Unlike Doxil, the myocet liposome does not have a polyethylene glycol coating and therefore does not result in the same prevalence of Hand-Foot Syndrome. The minimization of this side effect may allow 1: 1 substitution with doxorubicin in the same treatment regimen, thereby improving safety with no loss of efficacy.

DaunoXome® is a non-pegylated liposomal daunorubicin which is indicated in U.S for the first line treatment of Advanced AIDS-related Kaposi’s sarcoma [4].

Randomized trials comparing liposomal anthracyclines with conventional anthracyclines invariably present similar or higher efficacy with the liposomal anthracyclines. We performed a meta-analysis from nine randomized controlled trials of various tumors comparing the outcome and the adverse effects of conventional anthracyclines and liposome encapsulated or pegylated liposomal anthracyclines. To our knowledge this is the first meta-analysis comparing the safety of the conventional anthracyclines and the liposome encapsulated anthracyclines.

## Methods

We used a broad search strategy with special emphasis on randomized controlled trials. We used a variety of electronic databases, including MEDLINE via Pub MED, Ovid, and the Cochrane library.

First we identified key terms of the study drugs “liposomal doxorubicin”, “Doxil” “Myocet”, “Doxorubicin”, “Daunorubicin”, “Epirubicin”,“Mitoxantrone”,and “Idarubicin”. The key word “liposomal doxorubicin” compared with “Doxorubicin” was searched using the MeSH subheadings without any language barrier. The searches were combined with the key word search “randomized controlled trials”. 480 related articles were found, but only randomized controlled trials comparing liposomal doxorubicin and conventional anthracycline were selected for the meta-analysis. The selected randomized articles were again searched for related topics on the Pub MED database. We also searched unpublished studies with results through ‘clinical trials.gov’ and the American Society of Clinical Oncology (ASCO) website. We supplemented the searches by reviewing the bibliographies of key papers.

### Eligibility criteria

All randomized controlled trials that compared the efficacy of the liposome encapsulated doxorubicin with the conventional anthracyclines on any cancer with or without other chemotherapy were considered eligible for the analysis. The randomized controlled trials on pegylated liposomal doxorubicin (Doxil®/CAELYX™), non-pegylated liposomal doxorubicin (Myocet), and liposomal Daunorubicin (DaunoXome) pooled for the meta-analysis, irrespective of tumor types and stages. The controlled arms must include one of the conventional anthracyclines, epirubicin, daunorubicin, doxorubicin, idarubicin and mitoxantrone.

### Exclusion criteria

Non randomized trials were excluded. Randomized controlled trials with two different anthracyclines, but neither of them are a liposomal anthracycline were also excluded. Incomplete ongoing randomized trials with no published results were excluded as well.

### Data extraction

The following information about each trial was recorded: first author, journal name, year of publication, number of patients assigned, median age of the patients in each study, diagnosis, drug combinations and dose of treatment and the cumulative dose of anthracyclines. The adverse effects of the liposomal and conventional anthracyclines were analyzed in two arms. The variables for the adverse effects include cardiac toxicity, hematology toxicity, hand-foot syndrome or palmar plantar erythrodysesthesia (PPE), febrile neutropenia, alopecia, nausea and vomiting.

Different criteria were used to define the grade of toxicity. The majority of the trials (6 out of 9) used the National Cancer Institute-Common Toxicity Criteria (NCI-CTC). The remaining two trials used the WHO criteria for toxicity and one trial used South West Oncology Group (SWOG) toxicity scoring system. The differences on the toxicity grading of the variables were essentially unremarkable among the criteria [21].

The primary end points were the adverse effects: cardiac toxicity with congestive cardiac failure and significant reduction in the left ventricular ejection fraction (LVEF) were entered in separate arms. The information about the cumulative dose and cardiac toxicity was extrapolated in a separate excel sheet. Four out of the nine studies provided the data about the cumulative doses at which cardiac toxicity was developed. Analysis of cardiotoxicity included comparison of the proportion of patients in each treatment group who developed cardiotoxicity (by protocol specified cardiac event) at any time during the study, as well as comparison of the mean percentage change in LVEF from baseline.

The hematology toxicities (anemia, leukopenia, neutropenia and thrombocytopenia) were extracted to four variables representing the grades of toxicity. The information of the different grades of hematology toxicity was not available in two of the trials. We analyzed the number of the incidence of toxicity of any grade giving specific importance to the grade toxicity.

There is difference in the grading of alopecia between the WHO and NCI- CTC criteria. Alopecia, either partial or complete, was analyzed as one variable.

The nausea and vomiting were included as one variable. Two studies did not include the information about nausea and vomiting. Four out of 9 studies did not have grading for toxicity. Toxicity of all grades was therefore considered as a single variable.

### Statistical analysis

We performed the meta- analysis using the statistical software ‘Stata’ 10 version (statistics and Data created by Stata corp.). The existence of heterogeneity was tested using the Chi square statistics. The heterogeneity was quantified using the I-squared. For those variables with high heterogeneity(p<0.05 for I-squared analysis), the data were analyzed using the random effect model. Otherwise, fixed- effect model was used for the data analysis. We calculated the odds ratio (OR) with 95% confidence intervals (CIs) from the data extracted from the original studies separately. The overall ORs were calculated from the pooled data. We eliminated studies if the data were not available for a particular variable.

## Results

### Characteristics of the studies

Nine randomized controlled trials that enrolled a total of 2220 patients were selected for the meta-analysis (Table 1). 1112 patients were treated with liposome encapsulated anthracyclines, including 666 who received PLD, 330 who received LD, and 116 who received liposomal daunorubicin. 1108 patients received the conventional anthracyclines, including 80 who received epirubicin, and 1028 who received doxorubicin. The trials included four metastatic breast cancer trials, two multiple myeloma trials, two AIDS-related soft tissue sarcoma trials and one metastatic soft tissue sarcoma trials. Five of the studies used Pegylated liposomal formulation Doxil®/CAELYX and doxorubicin, four trials used non- pegylated liposomal formulations (Myocet™/Liposomal Daunorubicin) and conventional anthracyclines.

**Table 1:** Characteristics of eligible trials in the meta-analysis

**Trial**	**Year**	**Median age(LD)**	**Median age(D)**	**Tumor type**	**Total**	**Number of patients LD D**		**Control arm drug**	**Dose of anthracyclines**	**Liposomal anthracyclines**
Gill et al. [22]	1996	37	37	AIDS-KS	232	116	111	Doxorubicin	L dauno-40 mg/m2 Doxo-10 mg/m2	Lipo- Dauno
Northfelt et al. [23]	1998	36	38	AIDS-KS	258	133	125	Doxorubicin	PLD-20 mg/m2 D-20 mg/m2	PLD
Judson et al. [24]	2000	52	52	Metastatic soft tissue sarcoma	94	50	44	Doxorubicin	PLD-50 mg/m2 D- 75 mg/m2	PLD (CAELYX)
Harris et al. [8]	2001	58	58	MBC	224	108	116	Doxorubicin	LED- 75 mg Doxorubicin- 75 mg/m2	Myocet
Batist et al. [16]	2001	55	54	MBC	297	142	155	Doxorubicin	LED-60 mg/m2 Doxo-60 mg/m2	Myocet
O’Brien et al. [14]	2003	59	58	MBC	509	254	255	Doxorubicin	PLD-50 mg/m2 D- 60 mg/m2	PLD
Dimopoulos et al. [25]	2003	66	65	MM	259	132	127	Doxorubicin	doxil-40 mg doxo-9 mg/m2	Doxil bolus
Chan et al. [26]	2004	54	54	MBC	160	80	80	Epirubicin	LD-75 mg/m2 D-75 mg/m2	Myocet
Rifkin et al. [27]	2005	60	60	MM	192	97	95	Doxorubicin	Doxil-40 mg Doxo-9 mg/m2	Doxil

***Abbreviations*:***AIDS-KS*: Acquired immune deficiency syndrome- Kaposi Sarcoma; *D*: Doxorubicin; *LD*: Liposomal Doxorubicin; L *Dauno*: Liposomal Daunorubicin; *MBC*: Metastatic Breast Cancer; *MM*: Multiple Myeloma; *PLD*: Pegylated Liposomal Doxorubicin.

### Cardiotoxicity

**Figure 1:**
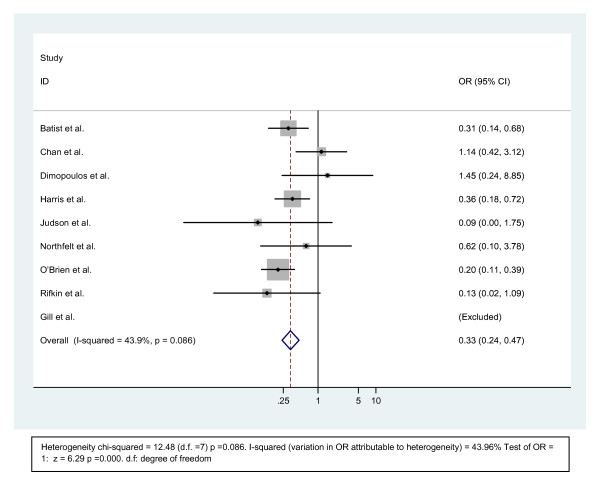
**Comparison of odds ratio in CHF.** The summary of OR wwas calculated using the fixed effect model. Squares are ORs of CHF for separate trials Horizontal lines through the scores represent 95% CIs. The diamond represents the overall OR of CHF from the meta-analysis and the corresponding 95% CIs. The studies that enrolled liposomal doxorubicin and conventional anthracyclines were separated into two groups for this analysis. **Abbreviations**: CI: Confidence interval; OR: Odd ratio; CHF: Congestive Heart Failure.

The cardiotoxicity (Figure 1) was higher with the doxorubicin group in five trails, whereas three other trials [14,25,26] did not show significant difference in cardiac toxicity in both doxorubicin and liposomal doxorubicin group. One trial did not report data on cardiac toxicity and therefore was not included for analysis of the cardiotoxicity [22]. The median cumulative dose of the doxorubicin in both forms was reported in six of the eight trials analyzed. The median cumulative dose is higher with the liposomal formulation in three of the trials [22,23,27], lower in one trial [14] and same in two trials [16,24].

Three studies [14,16,26] compared the CHF or change in LVEF based on the cumulative dose of anthracyclines. In the study by Chan et al., the number of patients with decrease in LVEF under a cumulative dose of <450 mg/m^2^ were 5 and 8 for liposomal doxorubicin and epirubicin, respectively. The change in LV function and CHF above and below the cumulative dose of 450 mg/m^2^ was lower with liposomal doxorubicin in O’Brien [14] and Batist [16] studies. In the study by Batist et al., the estimated median cumulative life time dose for cardiac toxicity was 2220 mg/m^2^ for the liposomal group and 480 mg/m^2^ for the non liposomal group.

The odds ratio for the study conducted by Chan [26] and Dimopoulos [25] were 1.14 and 1.45, respectively, which is in favor of the non liposomal arm. The odds ratios of six trials were in favor of the liposomal arm. The pooled analyses were statistically significantly in favor of the liposomal arm compared with the conventional anthracyclines. The combined odds ratio for the pooled result is 0.335 (95% CI (0.238–0.471, p = 0.086).

### Palmar-plantar erythrodysesthesia (PPE)/hand-foot syndrome

**Figure 2:**
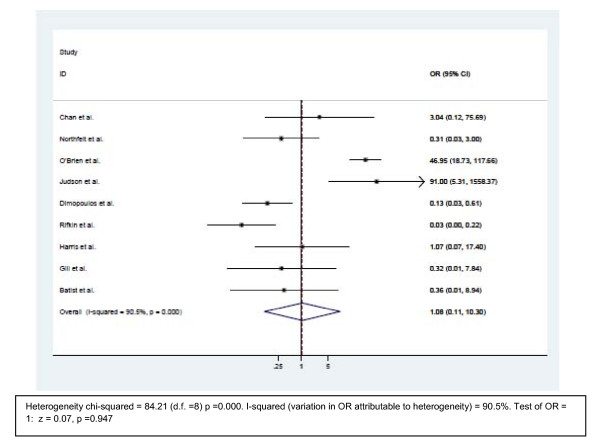
**Comparison of odds ratio in palmar-plantar erythrodysesthesia (PPE)/Hand foot syndrome (HFS).** The summary of OR was calculated using the random effect model. Squares are ORs of PPE/HFS for separate trials. Horizontal lines through the squares represent 95% CIs. The diamond represents the overall OR of PPE/HFS from the meta-analysis and the corresponding 95% CIs. The horizontal line with an arrow indicates the trial with highest OR. The studies that enrolled liposomal doxorubicin and conventional anthracyclines were separated into two groups for this analysis. **Abbreviations**: CI: Confidence interval; OR: Odd ratio; PPE.

Five trials used pegylated liposomal doxorubicin. Four trials used non pegylated liposomal anthracyclines (Figure 2). The odds ratios for PPE in five trials were in favor of the liposomal arm, the other four trials favored of the non-liposomal anthracyclines. The combined odds ratio of the trials were 1.08 (95% CI 0.11-10.30, p=0.947). Therefore, the overall result of the nine trials did not show statistically significant advantage of conventional anthracyclines over liposomal preparations in PPE events.

### Alopecia

**Figure 3:**
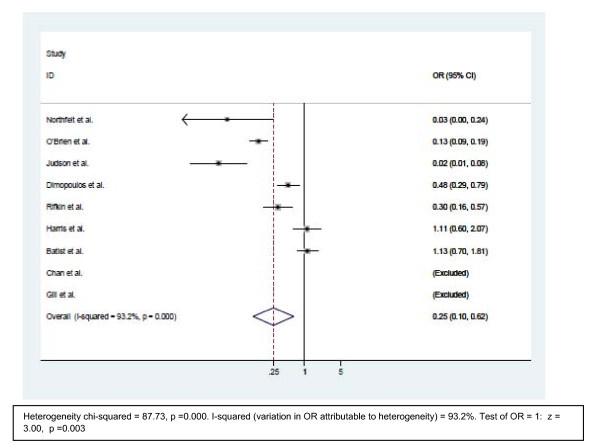
**Comparison of odds ratio in alopecia.** The summary of OR was calculated using the random effect model. Squares are ORs of alopecia for separate trials. Horizontal lines through the squares represent 95% CIs. The diamond represents the overall OR of alopecia from the meta-analysis and the corresponding 95% CIs. The horizontal line with an arrow indicates the trial with highest OR. The studies that enrolled liposomal doxorubicin and conventional anthracyclines were separated into two groups for this analysis. **Abbreviations**: CI: Confidence interval; OR: Odd ratio.

The incidence of alopecia (Figure 3) showed consistent results in 5 studies, whereas three studies showed no significant difference in the incidence of alopecia. The trials [14,23-25,27] showed significantly lower incidence of alopecia. All these trials compared PLD with doxorubicin. The three trials [8,16,26] that compared myocet with conventional anthracyclines failed to show a significant difference in the alopecia incidence. The overall odds ratio from the pooled analysis was 0.25 (95% CI 0.10–0.62, p = 0.003).

### Neutropenia

**Figure 4:**
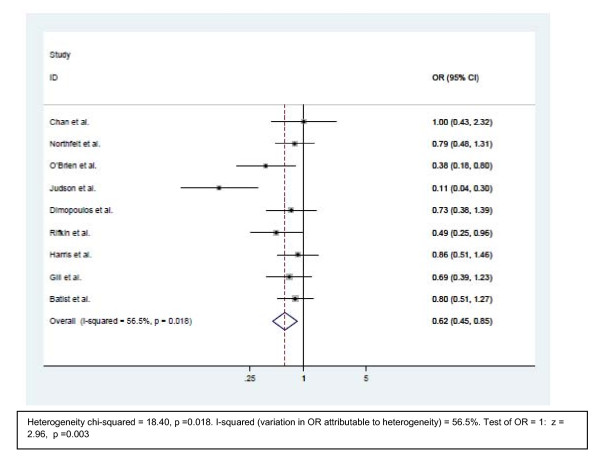
**Comparison of odds ratio in neutropenia.** The summary of OR was calculated using the random effect model. Squares are ORs of neutropenia for separate trials. Horizontal lines through the squares represent 95% CIs. The diamond represents the overall OR of neutropenia from the meta-analysis and the corresponding 95% CIs. The studies that enrolled liposomal doxorubicin and conventional anthracyclines were separated into two groups for this analysis. **Abbreviations**: CI: Confidence interval; OR: Odd ratio.

The incidence was counted for all grades of neutropenia. The odds ratios of eight of the nine trials are in favor of the liposomal doxorubicin (Figure 4). One study showed similar toxicity on both arms. The pooled result is in favor of the liposomal doxorubicin with an Odds ratio of 0.62 (95% CI is 0.45–0.85, p = 0.003).

### Febrile neutropenia

**Figure 5:**
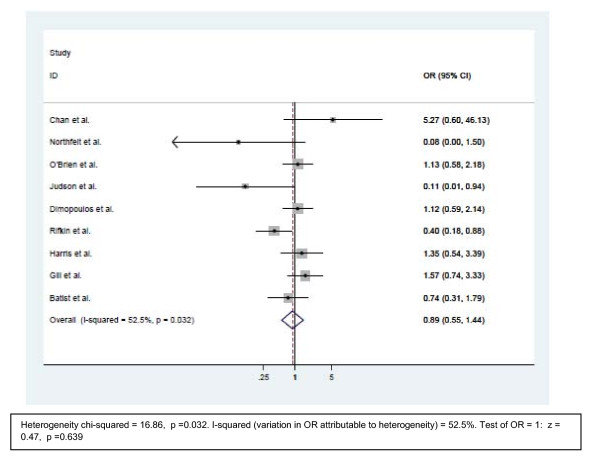
**Comparison of odds ratio in febrile neutropenia.** The summary of OR was calculated using the random effect model. Squares are ORs of febrile neutropenia for separate trials. Horizontal lines through the squares represent 95% CIs. The diamond represents the overall OR of Febrile Neutropenia from the meta-analysis and the corresponding 95% CIs. The horizontal line with an arrow indicates the trial with highest OR. The studies that enrolled liposomal doxorubicin and conventional anthracyclines were separated into two groups for this analysis. **Abbreviations**: CI: Confidence interval; OR: Odd ratio.

The odds ratios of all 9 studies were analyzed. The odds ratios of Chan, Dimpoulos, Gill, Harris and Northfelt studies were in favor of the non liposomal anthracyclines. The odds ratios of Batist, Judson, Northfelt and Rifkin studies were in favor of the liposomal arm. The overall OR for the pooled analysis of the nine trials was 0.89 (95% CI 0.55–1.44, p = 0.639), making both arms essentially similar adverse events of febrile neutropenia (Figure 5).

The other hematological toxicities anemia and thrombocytopenia were also in favor of the liposomal arm. The odd ratios of anemia and thrombocytopenia were 0.89(CI; 0.71–1.12) and 0.87(CI; 0.61–1.25) respectively. The incidence of nausea and vomiting was less with the liposomal arm with an odd ratio of 0.79 (CI; 0.66–0.96).

## Discussion

This meta-analysis included nine randomized controlled trials comparing liposomal and conventional anthracyclines.

Previous studies [28] have shown that the incidence of anthracycline induced CHF is directly proportional to the cumulative dose of anthracyclines. Northfelt, O’Brien and Batist plotted the relationship of cumulative dose of liposomal anthracyclines and conventional anthracyclines [14,16,23]. In this meta-analysis, the odds ratio reveals the incidence of cardiotoxicity to be significantly lower with the liposomal anthracyclines. The heterogeneity of the studies was low and the I² showed moderate heterogeneity for the cardiac toxicity. This study therefore confirms that the liposomal anthracyclines offer an alternative to conventional anthracyclines for patients with previous history of cardiac disease, elderly patients, and prior use of anthracyclines who are at high risk to develop cardiac toxicity. The safety profile with high cumulative dose range of liposomal anthracyclines favors the use of liposomal anthracyclines on patients who were previously treated with anthracyclines. The cardiac safety profile of the liposomal formulations of anthracyclines suggests the potential to use it in combination with trastuzumab in HER2 positive breast cancer.

The incidence of hematological toxicity of all grades was lower with liposomal anthracyclines. The incidences of neutropenia were significantly lower with liposomal anthracyclines, although there were no significant differences in febrile neutropenia. The lower incidence of myelosuppression makes the liposomal anthracyclines particularly more desirable for elderly patients.

PPE is a dose limiting toxicity of pegylated liposomal doxorubicin, doxil, which is the only liposomal doxorubicin approved in USA, even though the meta-analysis did not show significant differences in PPE.

The main limitations of the study are the heterogeneity of study groups. Though there was no difference within the study in each group, there were variations among different studies based on various factors. The primary cancer treated was metastatic breast cancer in four trials, multiple myeloma in two trials, AIDS related Kaposi sarcoma in two trials and metastatic soft tissue sarcoma in one trial. Due to the different tumor types, the study drugs varied in the dose, frequency and number of treatment. However, the intergroup variations have a limited effect on our meta-analysis due to the fact that each trial is randomized and well controlled. There are no variations among the two groups within the studies. The existence of heterogeneity among the study group was evaluated using the chi-squared analysis. The extent of heterogeneity was assessed using the I-squared analysis. Moderate to high heterogeneity was noted among the study groups. To minimize the bias, we used random effects models for the studies with high heterogeneity, as recommended and performed by many statisticians and meta-analysis publications [29-31].

## Conclusions

Liposomal doxorubicin and pegylated liposomal doxorubicin demonstrated favorable toxicity profiles with better cardiac safety and less myelosuppression, alopecia, nausea and vomiting compared with the conventional antracyclines. The better therapeutic index of liposomal anthracyclines without compromising the efficacy makes it a favorable choice over conventional anthracyclines in elderly patients, patients with risk factors for cardiac disease and patients with prior use of anthracylines.

## Competing interests

The authors have no relevant conflict of interests.

## Authors’ contributions

SMR and DL participated in concept design, data collection and analysis, drafting and critically revising the manuscript. MR participated in data analysis and figure preparation. BL, GL and GW participated in reference preparation and formatting. All authors read and approved the final manuscript.
